# The Relationship Between Tumor Volume and Timing of Post-resection Stereotactic Radiosurgery to Maximize Local Control: A Critical Review

**DOI:** 10.7759/cureus.5762

**Published:** 2019-09-25

**Authors:** Melissa Yuan, Eltion Behrami, Susan Pannullo, Theodore H Schwartz, A. Gabriella Wernicke

**Affiliations:** 1 Neurological Surgery, NewYork-Presbyterian/Weill Cornell Medical Center, New York, USA; 2 Radiation Oncology, NewYork-Presbyterian/Weill Cornell Medical Center, New York, USA; 3 Neurological Surgery, NewYork-Presbyterian/Weill Cornell Medical Center, New York, USA; 4 Neurology, NewYork-Presbyterian/Weill Cornell Medical Center, New York, USA

**Keywords:** brain metastasis, radiation, local control, stereotactic radiosurgery

## Abstract

After maximally safe neurosurgical resection of brain metastases, stereotactic radiosurgery (SRS) is now recommended as an alternative to whole-brain radiation therapy (WBRT), which has been associated with cognitive decline. One complicating factor associated with SRS is that postoperative cavity dynamics can change dramatically, creating significant variability in the recommended timing of SRS. While SRS has been shown to improve local control (LC) in smaller tumor cavities, achieving excellent LC rates still remains a challenge in larger ones. Furthermore, factors predicting the optimal timing of SRS in relation to the cavity size need to be defined and implemented. Variables such as the delay between postoperative MRI and treatment are critical but poorly understood. One potential treatment option that may improve outcomes is brachytherapy, but the widespread implementation of this technique has been slow. This critical review analyzes the relationship between preoperative tumor volume, resection cavity size, and timing of SRS and explores how these variables must be understood in order to achieve the highest LC possible.

## Introduction and background

Brain metastases may produce tumor mass effect that causes undesirable sequelae including seizures, nausea and vomiting, mental status changes, and other focal neurological defects [1]. Metastases to the brain are typically surgically resected in patients who have three or fewer brain metastases, or who have large tumors causing significant symptoms [2]. The currently accepted standard of care for post-resection treatment is whole-brain radiation therapy (WBRT). WBRT, despite its success in achieving local control (LC) of ~90%, has been associated with intolerably high toxic exposure to the brain and, consequently, decreased cognition and quality of life [3,4].

Stereotactic radiosurgery (SRS) is a method of delivering high-intensity, focused radiation with the surrounding normal tissue receiving a much lower dose of radiation than the resection cavity itself [5,6]. While there is a good deal of promising evidence that SRS can improve LC [5] while sparing the patient toxicity associated with WBRT, it remains difficult to accurately time and target SRS to the resection cavity size, especially in the setting of a larger cavity size/volume. This may be the reason that in some studies, long-term LC has been found to be worse with cavity SRS compared to post-resection WBRT [7,8]. Even so, many studies have shown comparable rates of LC with SRS compared to WBRT for resected brain metastases [7,9-14].

Currently, MRI is used to study the postoperative resection cavity of brain metastases for SRS planning. Part of this planning includes assigning margins to the cavity borders for the administration of the radiation. The challenge is that resection cavities are dynamic and often change unpredictably in shape and size during the interval between the planning of MRI and administering of SRS. This shift in resection cavity dimensions negatively impacts SRS's ability to optimally target areas of residual metastatic tissue while avoiding irradiation of normal tissue.

There have been several studies, both retrospective and prospective, detailing the shifts in resection cavity dimensions at various time points after surgery. In this review, we aim to define the postsurgical resection cavity changes in order to best inform SRS timing to optimize LC, survival time, and quality of life of patients.

## Review

Pubmed and Google Scholar from 2009 to 2019 were searched for articles pertaining to postoperative resection-cavity dynamics. Each study was evaluated for the number of patients, cavity-size change findings, cavity size and its influence on LC, and length of follow-up in addition to proposed explanations for observed results. Finally, seven studies that provided data on cavity dynamics after brain metastasis resection within the past 10 years were included.

Post-resection cavity-size changes: does the cavity shrink or grow after surgery?

The currently available literature indicates that resection cavities are dynamic after surgery. The majority of studies have found that, on average, resection cavities decrease in size postoperatively [2,15-18].

Specifically, Ahmed et al. found that 58% of post-resection cavities decreased in volume within one month of the procedure [16]. In this sample, 23 out of 39 cavities decreased by >10% of their initial dimensions after one month [16]. The authors demonstrated that greater postoperative edema was positively correlated with the subsequent cavity-size decrease in the month after surgery [16]. They found one interesting outlier: a patient with an edema of >15 mm who showed a <10% decrease in the cavity volume in a follow-up scan in the month after the surgery (since the size of edema was above the postoperative threshold, the imaging should have shown a larger percentage decrease in the cavity volume). However, this follow-up scan was conducted only 11 days after the resection [16]. The authors mention that this outlier patient then had a later MRI at 45 days after surgery, which demonstrated a 27% decrease in cavity size from the initial postoperative MRI, leading them to conclude that cavities do show a greater decrease in volume given more time [16]. However, as this study did not directly aim to examine cavity dynamics, they did not quantify how cavities across the sample changed over time.

In contrast to Ahmed et al.’s results, Shah et al. found that post-resection edema was not associated with cavity-volume decrease [16,18]. Across the sample, Shah et al. demonstrated a 43% mean decrease in cavity size [18]. This study only had volume data at the immediate postoperative MRI and the planning MRI, with an average of 41 days between [18]. Thus, while the authors were able to determine that patients who underwent imaging within one month of their initial postoperative MRI had a mean cavity-volume decrease of 13%, and those who underwent imaging more than one month after the initial MRI showed a decrease of 61% [probability value (p) =0.0003], they were unable to follow the cavity dynamics of each patient over the follow-up period.

Wernicke et al. found that in 30 patients who had had brain metastasis resection, the majority of the patients had cavity-volume decrease with a median cavity shrinkage of 84.8% at an average follow-up of 50 days [19]. Within each time period, the percentage decrease in size was -48% from the immediate postoperative MRI to the first follow-up; -68% from the immediate postoperative MRI to the second follow-up; and -85% from the immediate postoperative MRI to the third follow-up [19]. These percentage changes in cavity volume were statistically significant invariably (p = <0.001), showing that the cavities steadily continued their decrease in volume after resection.

Scharl et al. found an average cavity-volume reduction of 23.4% at a median of 23 postoperative days [20]. In line with Wernicke et al.’s results, this study showed a consistent decrease in cavity volume from the first postoperative MRI throughout the follow-up period [20]. The greatest reduction in cavity volume (-23.4%) occurred between the immediate postoperative MRI and the planning MRI (p = <0.01). Across the sample, 79.1% of the cavities decreased in volume and 3.5% were stable in size [20].

Alghamdi et al. found a significant overall decrease in cavity volumes, from an average preoperative tumor volume of 16 cm^3^ to a cavity volume of 12.4 cm^3^ at one month (p = 0.03) [2]. Across this sample, the average cavity volume reduction was 22.5% [2]. This study also found that within the early interval (<21 days), cavity size increased an average of 58.6% relative to the preoperative tumor volume, but decreased by 5% and 7% in the intermediate (21-41 days) and late (>42 days) intervals, respectively [2].

Predictors of differing rates of cavity-size changes

Regarding predictors of changes in cavity size, larger preoperative tumors and dural involvement were both found to be associated with greater decreases in volume [2]. In particular, Alghambdi et al. found that resection cavities of larger tumors (≥3 cm) had a greater volume reduction (16.3%) than smaller tumors (5.7%) at a median of 30 days after resection. Moreover, a greater proportion of the larger tumors vs. the smaller tumors showed considerable volume reduction: 72% of tumors that are >3 cm decreased in size, while only 44% of tumors that are <3 cm decreased in size [2].

Atalar et al. found compatible results, as cavities that were larger immediately after the surgery (tumors of >4.2 cm^3^) had a decrease in volume by 35% while smaller cavities (tumors of <4.2 cm^3^) actually increased in volume; these smaller cavities had an increase of 46% in volume within the first three days after surgery [15]. There was no significant change in cavity size for neither larger nor small cavities between the third day and 33rd day after surgery [15], which contrasts with the finding above of continued tumor size decrease even weeks after resection [2].

Larger tumor cavities being associated with a greater decrease in size was also seen by Jarvis et al. [21]. In this study, the authors found a 47% volume decrease in larger (>3 cm) cavities, a decrease of 25.8% for smaller (2.1 cm-3 cm) preoperative tumor cavities, and an increase of 56% in smaller (<2 cm) volume cavities at a median of 29.8 days after resection [21].

In contrast, Scharl et al. found that while, on average, all cavities across the sample decreased in size, smaller cavities tended to experience greater shrinkage. In other words, the initial cavity size was negatively correlated with the proportional cavity shrinkage between post-resection and planning MRI, which contrasts with the other findings that initial cavity size was positively correlated with cavity shrinkage [2,15,20,21]. In this study, the mean cavity size decreased over time from the immediate postoperative MRI to follow-up MRIs. The greatest reduction in cavity volume, an average of -23.4%, occurred between the immediate postoperative MRI and the SRS planning MRI (an average of 25 days; p = <0.01). Across the entire sample, between the immediate postoperative MRI and planning MRI, the cavity volume decreased in 79.1% of the sample, remained stable in 3.5%, and increased in 17.4% [20]. The mean percentage volume change over a median of 41 days was reflected as a reduction of -28.5% for cavities with a volume <10 cm^3^, and -13.3% for cavities with a volume of ≥10 cm^3^ as measured in the first postoperative MRI.

Regarding possible mechanisms of the observed results, Wernicke et al. postulated that the majority of cavity shrinkage was largely due to the initial collapse of brain tissue into the resection cavity [19]. Ahmed et al. suggested that the observed early cavity volume change was mostly due to vasogenic edema [16]. Conversely, Jarvis et al. examined the MRIs of patients with cavity-volume increase given their unexpected results of the cavity size and found that, on average, cavities increase in size. The underlying reason behind the increase in the size of the 13 cavities was the progression of local disease in two patients, accumulation of fluid or blood products in 9 patients, and nonspecific postsurgical enhancement around the cavity in two patients [21]. The changes in resection cavity dynamics found in each study are summarized below (Table 1).

**Table 1:** Changes in resection cavity size in relation to time during the postoperative period m: months, d: days

Reference	Study type	Number of patients	Number of resection cavities	Length of cavity size follow- up (mean/longest)	Early cavity-size change (post-op to Week 3)	Intermediate cavity-size change (week 3-week 6)	Late cavity-size change (week 6-later)	Overall resection cavity-size change as reported in the study
Ahmed et al., 2014 [16]	Prospective	37	39	1 m/1 m	Shrinkage	Shrinkage	No significant change	58% of cavities decreased in volume
Alghamdi et al., 2018 [2]	Prospective	59	61	1 m/3.6 m	No significant change	Shrinkage	Smaller cavities: shrinkage. Larger cavities: no significant change	Average cavity volume reduction of 22.5%.
Atalar et al., 2012 [15]	Prospective	63	68	20 d/33 d	No significant change past day 3 (days 1-3 had significant shrinkage)	No significant change	No significant change	Cavity volume did not change significantly
Jarvis et al., 2012 [21]	Retrospective	41	43	23.9 d/104 d	Shrinkage	No significant change	No significant change	20 cavities (46.5%) were stable, 10 cavities (23.3%) decreased in volume, 13 cavities (30.2%) increased in volume
Scharl et al., 2018 [20]	Retrospective	57	57	31 d/122 d	Shrinkage	Shrinkage	Shrinkage	Average cavity volume reduction of 23.4%. 79% of cavities had decrease in volume
Shah et al., 2016 [18]	Retrospective	21	21	41 d/Unknown	Shrinkage	Shrinkage	Shrinkage	90% of cavities had a decrease in volume
Wernicke et al., 2016 [17]	Prospective	30	30	Unknown/140 d	Shrinkage	Shrinkage	Shrinkage	Median cavity shrinkage of 84.8%

What is the optimal timing for the irradiation of large resected brain metastases with SRS?

A number of studies have shown that the resection cavity volume decreases over time; however, the time at which the greatest decrease occurred differed somewhat between the findings [2,16-18,20-22]. Thus, in this section, we will examine the specific time points at which there was a significant volume change.

Alghambdi et al. found that larger tumor cavities decreased in volume, while small tumor cavities initially (within the first 21 days) increased in volume [2]. Over the entire sample, the cavities, on average, increased in size relative to the original tumor size by 59% within the first 21 days, decreased by 5% between 22-41 days, and decreased by 11% after 42 days [2]. For small cavities, the authors found that the post-resection cavity was largest at the time of the immediate postoperative MRI and decreased at all follow-up time points for larger tumors, while cavity volume of the small tumors decreased after 21 or more postoperative days. They also found that large tumors showed a significant relative decrease in volume specifically during the intermediate postoperative period (between 22-42 postoperative days) [2].

In another study, Wernicke et al. found that postoperative resection cavities continuously decreased in size, when measured on day 1, 30 days, and 90 days after surgery, respectively [17]. The cavity shrinkage between each of these time points was statistically significant (time point 1: p = .002; time point 2: p = .001; time point 3: p = <.001), indicating that the decrease in cavity size continued consistently throughout the three-month period.

Shah et al.’s findings supported this pattern of results: significantly greater constriction occurred for patients with a longer time between MRIs (mean 61% reduction in volume) compared to those with an interval of <1 month between immediate postoperative MRI and planning MRI (13% reduction in volume). Across the entire sample, the tumor cavity shrank by an average of 43% at a median of 41 days after the initial postoperative MRI.

While Scharl et al. and Ahmed et al. also found decreased cavity size over time, they suggested that the most significant cavity-size changes occurred in the earliest postoperative period, specifically within 31 days [16,20]. Atalar et al. also found that the most significant cavity shrinkage occurred early, but within an even shorter timeframe; they found that the majority of cavities changed volume within 3 postoperative days, but did not change significantly from 3-33 days [15].

Jarvis et al. found that nearly 50% of resection cavities did not significantly increase or decrease in volume (defined as a change of ≤2 cm^3 ^), while 23% showed cavity shrinkage and 30% showed cavity expansion [21]. The mean time between initial postoperative and pre-SRS scans was 24 days (range: 2-104). However, Jarvis et al. did not report the timing of cavity shrinkage; thus, it is not possible to ascertain when these changes in volume occurred [21]. However, the authors did state that for the patients that showed an increase in cavity size, the increase was greatest during the interval between the MRI-2 and MRI-3, which occurred at an average on day 19.1 after the surgery (range: 4-76 days) [21].

**Figure 1:**
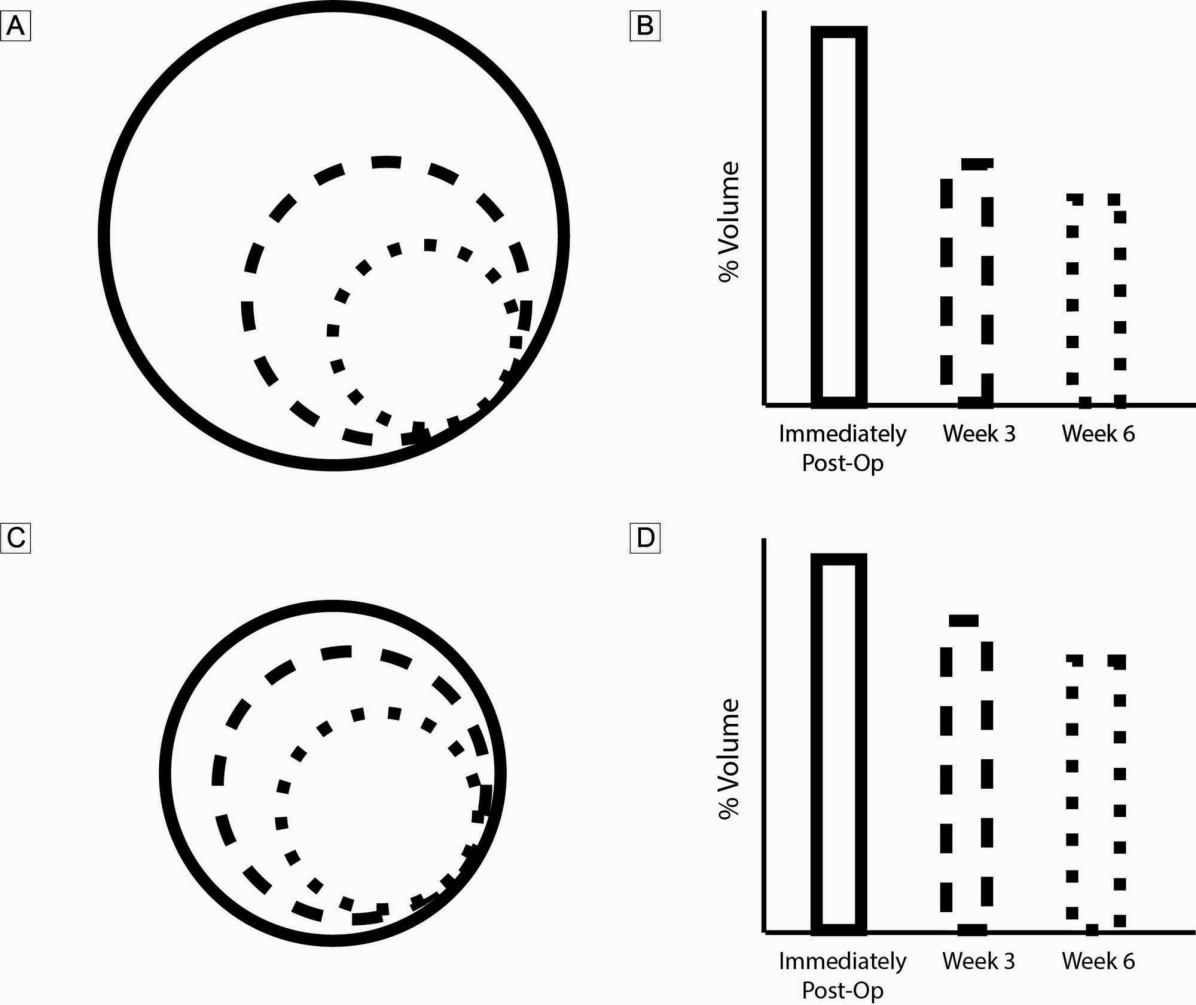
Schematic and estimated volume (%) of cavity-size changes over time A, B: large cavities (>3cm in diameter); C, D: small cavities (<3cm in diameter)

We present below a schematic of the changes in the postoperative cavity size for large and small tumors (Figure 1).

 

What are the causes of decreased local control with SRS?

One factor that predicts worse LC with SRS vs. WBRT after resection is tumor size. Several studies have found a negative correlation between increasing preoperative tumor size and a significantly diminished rate of LC where treatment failure is more likely in larger tumors [22-24]. Preoperative tumor sizes of greater than 3 cm have been found to have reduced LC over a range of 40% to ~70% [22,23].

The first prospective phase II trial that evaluated postoperative SRS found that preoperative tumors of >3.0 cm with superficial meningeal involvement, as well as infratentorial tumors, had higher rates of local failure [23]. Across the entire sample, the rate of local failure was 22% at a median follow-up of 12 months (range 1-94.1 months) [23]. Tumors of >3 cm had a 39.1% rate of local failure, while tumors of <3 cm had only a 7.5% rate of local failure at 12 months. As has been shown in other studies, meningeal involvement also predicted worse LC. In this study, the worst rates of LC were seen in large tumors (>3 cm) with superficial dural or pial involvement, with a 53.3% rate of local failure at 12 months [23].

Hartford et al.’s study, with a median follow-up of 9.3 months (range 1.1-61.4 months), also demonstrated that postoperative SRS led to decreased rates of LC in larger tumors vs. smaller tumors [23]. Univariate analysis found that preoperative tumor size of >3 cm predicted a significantly shorter time to local recurrence, and that preoperative tumor size of >2 cm also predicted a significantly shorter time to local recurrence, in addition to shorter time to distant recurrence and intracranial recurrence [23]. Lastly, Jensen et al. found on multivariate analysis that preoperative tumor diameter of >3 cm was associated with a 13.6 times greater risk of local failure compared to patients with tumor diameters of ≤3 cm at one year [hazard ratio (HR) = 13.6, p = 0.012] [25].

An earlier study had shown that while LC was achieved in 94% of the sample using postoperative SRS, there were three cases of tumor recurrence at the surgical site [14]. Interestingly, the treated volumes for the resection cavities in these cases (15.5, 18.4, and 21.1 cm^3^) were significantly larger than the volumes treated in the remaining cavities, leading the authors to conclude that patients who underwent radiosurgery to larger resection cavities had a higher likelihood of tumor recurrence [14].

Even for SRS to the tumor without surgical resection, greater tumor volume predicts worse LC [26]. That is, a tumor volume of ≥2 cm^3^ significantly predicted worse LC on univariate analysis (p = <0.001). Local tumor control was 97% for lesions of <2 cm^3^ at one year after SRS compared with 75% for lesions of ≥2 cm^3^ (p = <0.001). This was also demonstrated in an earlier study by Vogelbaum et al., which found that brain metastases larger than 2 cm were less effectively controlled than smaller tumors, though they did not report rates of local failure for different tumor sizes [27].

Another factor that is associated with worse LC by SRS is time between surgery and initiation of SRS. As noted, the volume of the tumor cavity can change significantly in the days and weeks after resection. Thus, if this change in volume is unaccounted for or incompletely accounted for as estimated by the most recent MRI before radiation treatment, rates of treatment failure increase [11,16,24,28]. This supports the findings that LC is worse with longer time between surgery and the initiation of treatment; that is, there is a greater risk of treatment failure with increasing delay after resection [23,28,29], which is hypothesized to be in part due to changes in cavity dimensions.

One retrospective study of 103 resection cavities in 100 patients found that SRS performed more than three weeks after resection was associated with greater probability of local recurrence, leading the authors to recommend that SRS should be performed as early as possible after surgery [30]. In this study, the authors used univariate analysis to find that longer surgery-to-SRS delay is a risk factor for recurrence [HR: 1.625; 95% confidence interval (CI): 1.183-2.231; p = 0.003]. They used Kaplan-Meier analysis to find that the maximum delay that best predicted LC was administering SRS within three weeks or earlier after surgery [30]. In this study, the overall rate of LC was 73% across the entire sample at 12 months. Stratifying this by time, the group of patients who received SRS less than three weeks after surgery had an 87% rate of LC at 12 months, while the group of patients who received SRS more than three weeks after surgery had a 61% rate of LC at 12 months [30]. This difference in rates of LC remained throughout follow-up; at 36 months, the group that received SRS less than three weeks after surgery had a 72% rate of LC compared to 46% for patients who received SRS more than three weeks after surgery [30].

A similar finding was replicated in another study by Strauss et al [28]. In this study, the overall LC rate was 84% at one year and 79% at two years [28]. As in the study by Iorio-Morin et al., Strauss et al. found that increasing delay between surgery and SRS was directly correlated with local failure using univariate log-rank regression on a sample of 102 brain metastasis cavities treated with postoperative SRS in 100 patients (HR: -1.46, p = 0.02) [28]. This study did not divide the patients into groups with differing times from surgery to SRS, using univariate regression instead. As a result, we do not have rates of LC for patients who received SRS more or less than three weeks after surgery for comparison to the previous study.

There is wide acceptance that radiation treatment to the resection cavity should not be unnecessarily delayed. However, there are different protocols between institutions as to the ideal time for SRS after surgery, with some groups recommending earlier than six weeks, and others recommending earlier than three weeks, with a general acceptance of around four weeks [11,16,25,28,30]. The Radiation Therapy Oncology Group (RTOG) has yet to provide exact guidelines regarding the optimal timing of postoperative SRS. However, a recent review recommends that based on current evidence, performing SRS within 2-3 weeks after surgery is the best option to allow the patient to recover surgically, without excessively delaying postoperative treatment [31]. This is based on recent evidence from Iorio-Morin et al. that a significant risk factor for decreased LC is a delay of greater than three weeks before surgery, and Patel et al., who found no significant cavity shrinkage after 2-3 weeks and concluded that SRS within 2-3 weeks is most appropriate [30,32].

Based on the current evidence showing the unpredictability of the exact pattern of change in resection cavity size over time, we can conclude that obtaining a planning MRI as close in time to treatment as possible should be prioritized [21,25,31]. This was supported by Ahmed et al.’s findings that more patients became eligible for SRS with increasing follow-up time due to cavity shrinkage; Jarvis et al.’s results showing unpredictable rates of cavity volume increase or decrease during follow up; and the numerous other studies showing continuous cavity-volume decrease after surgery [18-20]. For example, in the study by Ahmed et al., four cavities were >40mm on the immediate postoperative MRI. A tumor cavity of this size is generally considered a poor candidate for SRS [16]. However, the authors found that because all four of these large cavities decreased to be below SRS threshold of 30 mm diameter within their follow up time, then rescanning of all cavities, but especially the ones closest to the treatment cut-off threshold, as close to the planned treatment date may allow for more patients to be eligible for SRS treatment [16]. As the majority of studies showed a continuous cavity-size decrease over time, this would indicate that obtaining a planning MRI closer to the date of treatment would perhaps allow for a lower total dose of radiation to be used, given a smaller volume to irradiate [18-20,28].

Advantages and disadvantages of two focal radiation treatments

It has been shown that both SRS and intraoperatively implanted brachytherapy seeds lead to good disease control and decreased likelihood of recurrence, as the postoperative adjuvant irradiation can eliminate the microscopic disease that remains in the resection cavity [28,33,34]. However, given the different dynamics for large vs. small resection cavities at different points in time, it is important to understand which focal radiation treatment might work best in specific situations. To this end, we will review the advantages and disadvantages of SRS and intraoperative brachytherapy with volume-based implants in the management of brain metastases.

SRS is a non-invasive method of administering focused, convergent beams to deliver a high dose of radiation to an area of interest. Brachytherapy is another increasingly popular method of providing local high-dose radiation but requires a neurosurgical procedure with intraoperative placement of radio-isotopes [35]. One important consideration in choosing SRS over brachytherapy is the size of the tumor cavity; a preoperative tumor diameter of less than 3 cm is the commonly accepted size for consideration of treatment with SRS [1]. This is because SRS has shown inferior rates of control to larger resected tumor cavities in multiple studies. One large retrospective study found that larger tumors, when treated with SRS, had a shorter time to recurrence and symptomatology than smaller tumors treated with SRS [23]. Another study found that a preoperative tumor diameter of >3 cm predicted greater risk of local treatment failure [25]. This finding that SRS is less effective in establishing LC in larger tumors has been replicated numerous times [14,22,23].

Another reason that SRS is not deemed the best choice in larger tumors is the higher rate of radiation necrosis, which can occur in up to 37.8% of patients within one year, specifically after tumors of >1.5 cm in diameter are treated with the procedure [36]. This is most likely due to larger tumor resection cavities increasing the radiation burden on surrounding normal tissue when SRS is implemented, thus increasing the risk of edema and necrosis [5]. Conversely, larger cavities treated with intraoperative cesium-131 (Cs-131) implants have demonstrated excellent LC [17,19]. Previous trials with iodine-125 brachytherapy have also demonstrated very good LC in both large and small resected tumor cavities [37,38]. Interestingly, multidose SRS (9Gy x 3) has also shown good LC of 93% at one year for large (>3 cm) brain metastases [39], although this has not been commonly replicated.

An advantage of SRS is that it has been shown to effectively treat multiple metastatic foci. While SRS can be used for up to three resection cavities of CNS metastases, brachytherapy generally has only been shown to be effective in one resection cavity [19,37]. However, an advantage of brachytherapy is that it can be more readily applied to irregularly shaped cavities, while SRS is most suited to round cavities [11,25].

**Figure 2:**
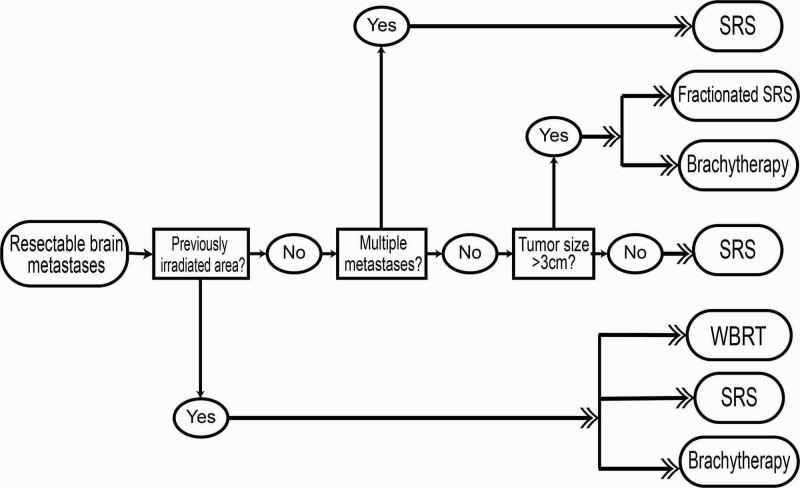
flow-chart algorithm detailing situations in which different radiation techniques are preferable

One particular advantage recently documented with brachytherapy is that seeds can be implanted in such a way as to maintain cavity volume. Wernicke et al. implanted Cs-131 seeds with fibrin glue, and this maintained cavity volume, thus avoiding cavity-volume fluctuation [17]. Significantly, this decreased fluctuation led to less variation in the proximity of the radiation seeds to each other and to normal tissue, thus stabilizing dosage strength [17,35]. Brachytherapy such as Cs-131 is also advantageous over SRS in cases of recurrent brain metastases in brains that have already been irradiated, as repeat irradiation greatly increases the risk of radiation necrosis [36]. This is because brachytherapy has the advantage of a steep dose fall-off outside of the immediate area of the resection cavity, leading to less irradiation of normal tissue [19,38]. Also, it has been hypothesized that immediate implantation of brachytherapy seeds may improve LC by counteracting the tumor-cell proliferation and dissemination caused by the surgical manipulation of the microenvironment during resection [38]. We present an algorithm detailing situations in which different radiation techniques are preferable (Figure 2). 

## Conclusions

In conclusion, we have found that current evidence supports a general trend of cavity-site decrease after surgery, and larger tumor cavities generally have a greater average percent decrease compared to smaller cavities. To further apply this knowledge of cavity dynamics to treatment, we have reviewed two focal radiation treatments: SRS and brachytherapy. SRS is advantageous in smaller, more uniform tumor cavities, in addition to situations of multiple resection cavities, while intraoperative brachytherapy may be used for larger (>3 cm), more irregularly shaped tumor cavities. Our review also indicates that resection followed by SRS of smaller metastases is likely to benefit the patient; as the resection cavity shrinks, the volume of normal tissue that may be irradiated decreases. As it was shown that smaller resection cavities have a longer duration of decrease compared to larger tumors, it is possible that delaying radiosurgery on smaller metastases may be beneficial. We hope that this knowledge can be useful in deciding on the type and timing of post-resection radiosurgery for brain metastases.
